# Importance of pre-analytical steps for transcriptome and RT-qPCR analyses in the context of the phase II randomised multicentre trial REMAGUS02 of neoadjuvant chemotherapy in breast cancer patients

**DOI:** 10.1186/1471-2407-11-215

**Published:** 2011-06-01

**Authors:** Patricia de Cremoux, Fabien Valet, David Gentien, Jacqueline Lehmann-Che, Véronique Scott, Carine Tran-Perennou, Catherine Barbaroux, Nicolas Servant, Sophie Vacher, Brigitte Sigal-Zafrani, Marie-Christine Mathieu, Philippe Bertheau, Jean-Marc Guinebretière, Bernard Asselain, Michel Marty, Frédérique Spyratos

**Affiliations:** 1 Department of tumour Biology, Institut Curie, Paris 75005, France; 2 Department of Biostatistics, Institut Curie, Paris 75005, France; 3 Inserm U900, Institut Curie, Paris 75005, France; 4 Translational Research Department, Institut Curie, Paris 75005, France; 5 Department of Biochemistry, AP-HP, Saint-Louis Hospital, University Paris Diderot, Paris 75010, France; 6 Translational Research Laboratory, Institut Gustave Roussy, Villejuif 94805, France; 7 Department of Oncogenetic, Institut Curie, Hôpital René Huguenin, Saint Cloud 92210, France; 8 Department of Pathology, Institut Gustave Roussy, Villejuif 94805, France; 9 Department of Pathology, AP-HP, Saint-Louis Hospital, University Paris Diderot, Paris 75010, France; 10 Department of Pathology, Institut Curie, René Huguenin Hospital, Saint Cloud 92210, France; 11 Centre for Therapeutic Innovations in Oncology and Haematology, AP-HP, Saint-Louis Hospital, University Paris Diderot, Paris 75010, France

**Keywords:** neoadjuvant setting, transcriptome, RT-qPCR, breast cancer, quality criteria

## Abstract

**Background:**

Identification of predictive markers of response to treatment is a major objective in breast cancer. A major problem in clinical sampling is the variability of RNA templates, requiring accurate management of tumour material and subsequent analyses for future translation in clinical practice. Our aim was to establish the feasibility and reliability of high throughput RNA analysis in a prospective trial.

**Methods:**

This study was conducted on RNA from initial biopsies, in a prospective trial of neoadjuvant chemotherapy in 327 patients with inoperable breast cancer. Four independent centres included patients and samples. Human U133 GeneChips plus 2.0 arrays for transcriptome analysis and quantitative RT-qPCR of 45 target genes and 6 reference genes were analysed on total RNA.

**Results:**

Thirty seven samples were excluded because *i) *they contained less than 30% malignant cells, or *ii) *they provided RNA Integrity Number (RIN) of poor quality. Among the 290 remaining cases, taking into account strict quality control criteria initially defined to ensure good quality of sampling, 78% and 82% samples were eligible for transcriptome and RT-qPCR analyses, respectively. For RT-qPCR, efficiency was corrected by using standard curves for each gene and each plate. It was greater than 90% for all genes. Clustering analysis highlighted relevant breast cancer phenotypes for both techniques (ER+, PR+, HER2+, triple negative). Interestingly, clustering on trancriptome data also demonstrated a "centre effect", probably due to the sampling or extraction methods used in on of the centres. Conversely, the calibration of RT-qPCR analysis led to the centre effect withdrawing, allowing multicentre analysis of gene transcripts with high accuracy.

**Conclusions:**

Our data showed that strict quality criteria for RNA integrity assessment and well calibrated and standardized RT-qPCR allows multicentre analysis of genes transcripts with high accuracy in the clinical context. More stringent criteria are needed for transcriptome analysis for clinical applications.

## Background

Breast cancer is a clinically and biologically heterogeneous disease. Most breast cancer patients in whom primary systemic therapy is proposed are treated with anthracycline- and/or taxane-based regimens. However, no clearly validated predictive or prognostic factors are available to determine the best regimen to obtain a pathological complete response and improve survival in patients with breast carcinoma. Neoadjuvant chemotherapy is associated with the same survival benefits as adjuvant chemotherapy with increased breast-conserving surgery rates [1,2]. Furthermore, neoadjuvant chemotherapy represents an opportunity to correlate molecular variables in the initial biopsy with treatment response and to explore mechanisms of drug resistance. Identification of predictive markers of individual response to chemotherapy is a major challenge to ensure optimal treatment for patients. Microarray and quantitative reverse transcription polymerase chain reaction (RT-qPCR) technologies are complementary powerful tools to define gene expression profiles in breast carcinoma. However, further work is required to move these tools from research laboratories to clinical practice, mainly by improving standardization of the process and management of samples and derived material before analysis.

The neoadjuvant chemotherapy REMAGUS 02 (RO2, ISRCTN 10059974) [3] was designed to assess anti-tumor activity of sequential epirubicin/cyclophosphamide followed by docetaxel with the randomized addition of celecoxib in HER2 negative patients or trastuzumab in HER2 positive patients [3]. Four centres participated in this trial and frozen biopsies were mandatory for enrolment. An ancillary study was conducted prospectively in order to define predictors of response to chemotherapy

The aim of the present study was to present and discuss the importance of pre-analytical and analytical steps for transcriptome analysis (using Affymetrix U133A2) and for RT-qPCR of 45 target genes of interest in breast cancer in the context of this ancillary study.

## Methods

### REMAGUS 02 (RO2) study design

This study was conducted from May 2004 to October 2007 as a prospective ancillary study of a multicentre randomised phase II trial in 340 patients with stage II and III breast carcinoma, ineligible for breast-conserving surgery and treated with neoadjuvant chemotherapy (sequential three-weekly cycles of epirubicin (75 mg/m^2^)/cyclophosphamide (750 mg/m^2^) for 4 cycles followed by docetaxel (100 mg/m^2^) for 4 cycles. HER2-negative patients (n = 220) were randomised to concomitantly receive docetaxel, celecoxib (800 mg/day) during cycles 5 to 8, or placebo. HER2-positive patients, diagnosed by IHC and systematically confirmed by FISH analysis (n = 120), were randomised to concomitantly receive docetaxel, trastuzumab (8 mg/kg then 6 mg/kg every 3 weeks) during cycles 5 to 8, or placebo. Details and results of this trial have been previously published by Pierga *et al*. [3]. Four French centres, namely centres 1 to 4, were involved in this trial. All patients were informed and gave their signed consent to participate in the trial and the ancillary studies (French Ethics Committee n°03-55). The primary objective was pathological complete response (pCR), evaluated according to Chevalier's criteria [4]. Secondary objectives were to define genomic profiles of success (pCR) or failure of each type of treatment.

### Tissue samples

Availability of frozen tumour tissues from molecular studies was mandatory for inclusion in the trial. Breast biopsies were obtained by a 14-gauge core biopsy device (centres 1, 2, 3), or surgical biopsy (centre 4) prior to treatment with one specimen dedicated to standard pathological diagnosis, and two specimens to RNA extraction. Fully anonymized biopsy samples were used in accordance with each institution's ethical rules. Tumour biopsies were immediately snap frozen in RNAse-free conditions and stored at -80°C or in liquid nitrogen, at local sites. At the end of the trial, they were shipped in dry ice to the laboratories performing the various assays.

Tumour cellularity was evaluated on frozen sections of the biopsies dedicated to RNA extraction by local staff breast pathologists. Recordings criteria were defined at a consensus meeting prior to the study. The percentage of invasive and *in situ *malignant cells was recorded *versus *the amount of benign epithelial cells, stromal cells, inflammatory cells and necrosis. Semi-quantitative evaluation was performed. For patients in whom multiples biopsies were available, the biopsy with the highest invasive content was used for the analysis. Only samples containing at least 30% tumour cells were kept for further analysis.

### RNA isolation, quantification and qualification

Total RNA was processed at the end of the trial, in centres 1, 2 and 3, using a common TRIzol method according to the manufacturer's instructions (Invitrogen Corporation, Carlsbad, USA) followed by RNA clean-up using the NucleoSpin RNA II kit (Macherey-Nagel, Hoerdt, France). In centre 4, total RNA was extracted prospectively during the trial by using a two-round TRIzol method followed by the same clean-up.

The quantity and purity of extracted RNA were assessed by measuring absorbance at 230, 260 and 280 nm using a NanoDrop ND 1000 spectrophotometer (Wilmington, USA). Only samples with a 260/280 ratio between 1.8 and 2 and a 260/230 ratio greater than 1.6 were included in subsequent studies.

Evaluation of RNA integrity was performed using the Agilent Bioanalyzer 2100 microfluidics-based platform and the RNA 6000 Nano Lab Chips kit (Agilent, Santa Clara, CA, USA) in all centres. It was determined by the combination of the following criteria: 28 s/18 s ratio, RIN (RNA Integrity Number) and electrophoretic profile (level of degradation, flat or wavy baseline, DNA contamination, etc.).

Extracted total RNA was submitted to further analysis if the RIN was greater than 7 and 6 for transcriptome and RT-qPCR analyses, respectively. Three μg total RNA were kept for transcriptome analyses, 1 μg for RT-qPCR and 500 ng for the FASAY assay to detect p53 mutations (data will be presented in a separate paper). When an insufficient quantity of RNA material was available to perform the three analyses, priority was given to transcriptome analysis. All samples were tested for albumin DNA contaminants using an intronic albumin gene design in qPCR. No amplification of albumin DNA was observed in our series of samples (Ct > 35). Total RNA of human breast cancer cell lines T47D and MDA-MB 231 were used to calibrate reverse transcription and standardize real-time PCR.

### Transcriptome analysis

Analyses were performed at the Institut Curie Translational Research Department (Paris). Two micrograms of total RNA were reverse transcribed into cDNA. After synthesis of double-stranded cDNA, an *in vitro *transcription reaction was conducted overnight. Resulting amplified cRNA, labelled by means of biotinylated pseudo-uridine, was then purified. Experimental replicates (MAQC A sample, Universal Human Reference RNA, Stratagene, 740000-41) were included from the first step in each batch of target preparation to evaluate the reproducibility and batch effect of the whole RNA processing procedure [5]. In order to monitor the quality of targets before microarray hybridization, the Institut Curie Affymetrix platform established a quality control based on analysis of the cRNA profile: no more than 40% small fragments (36-500 nt); more than 20%. long fragments (> 1500 nt). For samples failing to satisfy these criteria, a Test3 (GeneChip^®^3 Array, Affymetrix) was used and only high quality cRNA samples from Test3 microarray assays were processed for human pan-genomic microarray hybridization, i.e. when backgrounds were less than 100 and the housekeeping gene 3'/5' ratio was less than 3 for GAPDH and less than 6 for β-actin.

Human Genechip U133 plus 2.0 microarray hybridization was performed with precisely 10 μg fragmented cRNA (35-200 nt), overnight at 45°C with shaking. The detailed protocol can be downloaded from:

http://www.affymetrix.com/support/downloads/manuals/expression_analysis_technical_manual.pdf. The hybridization mix was removed and stored at -80°C, while the microarray fluorescence was elicited using the Genechip^® ^Fluidics Station 450. The hybridized biotinylated-cRNA signal was amplified by successive reactions with phycoerythrin-conjugated streptavidin and biotin. The fluorescence signal intensity was measured with the GeneChip^® ^Scanner 3000 (1.56 μm resolution).

### Real-time RT-qPCR analysis

Analyses were performed at Institut Curie Molecular Pharmacology Unit and the Centre René Huguenin Oncogenetic Laboratory using a common RT-qPCR procedure.

First-strand cDNA synthesis was performed with 1 μg total RNA using Superscript II Reverse Transcriptase (Invitrogen Corporation) in a final volume of 20 μL, as previously described [6-8]. Quantitative PCR analysis was performed on 6.25 ng cDNA in duplicate. A 5 μL diluted sample of cDNA (6.25 ng) was added to 20 μL of the PCR mix. The thermal cycling conditions comprised an initial denaturation step at 95°C for 10 min, 45 cycles at 95°C for 15 sec, and either 60°C or 65°C depending on the target, for 1 min.

All PCR reactions were performed using the ABI Prism 7900 Sequence Detection System (Applied Biosystems Inc., Forster City, USA). The PCR Core reagent kit was used for systems with Taqman probes (Eurogentec, Liège, Belgium), and the Power SYBR Green PCR master Mix (Applied Biosystems Inc.) was used for systems without Taqman probe. Primers and fluorescent probes were designed from published sequences using Primer express software (Applied Biosystems Inc.). BLASTN searches against dbEST and nr (the nonredundant set of the GenBank sequence database) were performed to confirm the total gene specificity of the chosen nucleotide sequences and the absence of DNA polymorphisms. Target sequences were 60-120 long. Forty-five cancer-related target genes (Additional file 1) involved in the main signalling pathways associated with in breast cancer development were studied (nucleotide and probe sequences available on request). RPLPO, TATA Box binding protein (TBP), transferrin receptor (TFR), β-actin, β-glucuronidase (GUS), and GAPDH were used as endogenous reference genes. *Transferrin receptor-TFRC-5' (Hs00951086_m1)*, GAPDH-5' (Hs99999905_m1) and GUSB-3' (Hs99999908_m1) were obtained as Assays-on-Demand from Applied Biosystems. Human breast cancer cell lines T47D and MDA-MB 231 cDNA were used to generate 8 points standard curves for each gene [6,9]. Target quantities were normalized to each of the reference genes and to the median of the 6 reference genes and calibrated using the second point of each standard curve. Final results were expressed as N-fold differences in target gene expression relative to the reference genes and the calibrator and are expressed as [6]:

where E is the efficiency of PCR measured using the slope of the calibration curve, and Ct is the cycle threshold.

No Reverse-transcription Controls (NTC) were included in each batch of samples. Only cases with exploitable data obtained for the 6 reference genes and the 45 target genes were submitted to further statistical analysis (Additional file 1).

### Descriptive analysis and graphical representation RT-qPCR and transcriptome

We used R software [10] for descriptive analysis and graphical representations. Details on the functions used are given in Supplementary Methodology (Additional file 2).

In a first step, median estimates [min - max] of tumour cell percentage, RIN, 260/280 and 28 s/18 s ratios, and RNA quantities were provided to describe the distributions of quantitative characteristics of RNA used for both transcriptome and RT-qPCR analysis. Comparisons of reference genes Ct means between each of the four centres were performed using ANOVA (ANalysis Of VAriance). For each of the 6 reference genes, a global p-value of Ct mean's heterogeneity between all centres was calculated. In order to account for a possible center effect, a p-value of Ct mean's heterogeneity was also provided when excluding one centre from the ANOVA.

In a second step, a hierarchical clustering on the 239 patients was performed. Spearman correlations of the 45 E^ΔΔCt ^values between patients (taking each of the six reference genes or the median of the 6 reference genes Ct, as the reference gene) were proposed as similarity measures, and we used a Ward algorithm as the agglomerative criterion. Separation into groups was proposed on the basis of the structure of the dendrogram. Hormonal receptor status (ER+, PR+), HER2 status (HER2+), Histological Grade (EE grading system) [11], and the centre were indicated on the graphic for each patient.

Microarrays data provided by the four centres were normalized together using the GCRMA procedure [9]. Then, a hierarchical clustering on the 226 patients was performed. Spearman correlations of the 5000 probe sets showing the highest values of interquartile range (difference between the third and first quartiles) were used as similarity measures, and we used a Ward algorithm as the agglomerative criterion. Hormonal receptor status (ER+, PR+), HER2 status (HER2+), Histological Grade, and the centre were also highlighted for each patient.

## Results

**Figure 1:**
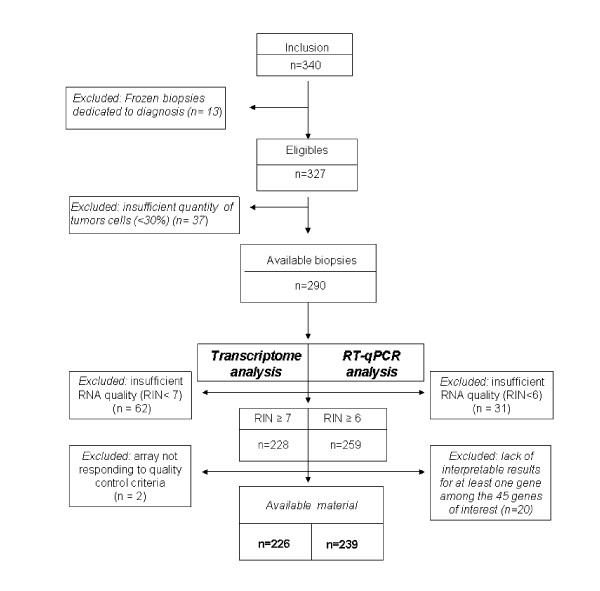
**Flow diagram of patients included and frozen tumors of samples available in REMAGUS 02 biological trial (biopsies)**.

Among the 340 pretreatment frozen biopsies, 13 (4%) initially dedicated to translational studies were used for diagnosis due to an insufficient quantity of tumour cells in the diagnostic biopsy and were therefore not included. Thirty-seven of the 327 biopsies (11%) were also excluded because they contained less then 30% of tumour cells. Consequently, 290 biopsies were eligible for total RNA extraction (Figure 1).

### RNA quantification and qualification

According to the qualification criteria defined for each analysis, 226/290 (78%) samples were available for transcriptome analysis and 239/290 (82%) samples were available for RT-qPCR. In both cases, we observed that the proportions of lobular and low grade carcinoma were significantly higher in excluded material (Additional file 3 and 4). The median RNA quantity for the 4 centres was 13.21 μg and 12.52 μg for transcriptome and RT-qPCR series respectively (Table 1 and Table 2). Median RIN values were equal to 8.40 both for transcriptome and RT-qPCR series (Table 1).

**Table 1:** Quantitative and qualitative characteristics of RNA used for transcriptome analysis: **median **[min - max]

Characteristics	Centre				All centres
		
	1	2	3	4	
**Tumour** **cells (%)**	60 [30 - 95]	60 [30 - 90]	50 [30 - 90]	70 [30 - 90]	60 [30 - 95]

**RIN**	8.40 [6.30 - 9.90]	9.10 [6.90 - 10.00]	8.10 [7.00 - 9.30]	8.30 [6.40 - 9.30]	8.40 [6.30- 10.00]

**260/280 ratio**	2.08 [1.95 - 2.13]	2.05 [1.72 - 2.17]	2.07 [2.02 - 2.12]	2.09 [1.94 - 2.14]	2.08 [1.72 - 2.17]

**28 s/18 s ratio**	1.60 [0.70 - 2.10]	1.70 [1.10 - 2.40]	1.40 [1.10 - 1.80]	1.30 [1.00 - 2.00]	1.60 [0.70 - 2.40]

**RNA (μg)**	16.91 [3.38 - 89.58]	15.98 [2.92 - 64.31]	12.58 [5.00 - 23.00]	6.87 [2.16 - 20.34]	13.21 [2.16 - 89.58]

**Table 2:** Quantitative and qualitative characteristics of RNA used for RT-qPCR analysis: **median **[min - max]

Characteristics	Centre				All centres
		
	1	2	3	4	
**Tumour** **cells (%)**	60 [30 - 95]	60 [30 - 90]	50 [30 - 90]	70 [30 - 90]	60 [30 - 95]

**RIN**	8.40 [6.10 - 9.90]	8.90 [6.70 - 10.0]	8.00 [6.00 - 9.30]	8.30 [6.40 - 9.30]	8.40 [6.00 - 10.00]

**260/280 ratio**	2.08 [1.99 - 2.13]	2.05 [1.72 - 2.17]	2.08 [2.02 - 2.15]	2.09 [1.94 - 2.14]	2.08 [1.72 - 2.17]

**28 s/18 s ratio**	1.60 [0.70 - 2.10]	1.70 [1.10 - 2..40]	1.35 [1.00 - 1.80]	1.30 [0.90 - 2.00]	1.60 [0.70 - 2.40]

**RNA (μg)**	16.77 [2.49 - 89.58]	11.02 [3.15 - 31.95]	13.92 [5.00 - 26.93]	6.90 [3.60 - 20.34]	12.52 [2.49 - 89.58]

### Transcriptome validation criteria

Microarray data are available on GEO with accession number GSE26639. Here is the link for access:

http://www.ncbi.nlm.nih.gov/geo/query/acc.cgi?token=lhynjakuucioojq&acc=GSE26639.

Among the 226 patients eligible for transcriptome analysis, the amplification yields were sufficient to prepare large quantities of cRNA (median: 68 μg) (Additional file 5). Amplified cRNA, controlled by electrophoresis, contained a median of 26% of small fragments and 41% of long fragments. Qualitative analysis of the hybridization signals using MAS5 summarization algorithm showed that all microarrays had a low background signal (median: 59.66). For all centres, the median percentage of present call was 50.04%, and the GAPDH and β-actin 5'/3' ratios were similar to those obtained on Test3 arrays (Additional file 6).

**Figure 2:**
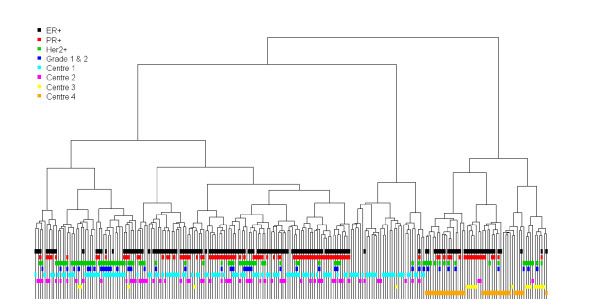
**Transcriptome analysis: Clustering of 226 patients based on the 5,000 probe sets having the highest Inter-Quartile Range values**.

Clustering analysis using the 5,000 most variable probes highlighted relevant breast cancer phenotypes: ER+, PR+, HER2+ and triple-negative tumours (Figure 2 andAdditional file 7). A centre effect was also observed, as illustrated by a cluster defined by patients of centre 4.

### Quantitative RT-qPCR validation criteria

**Figure 3:**
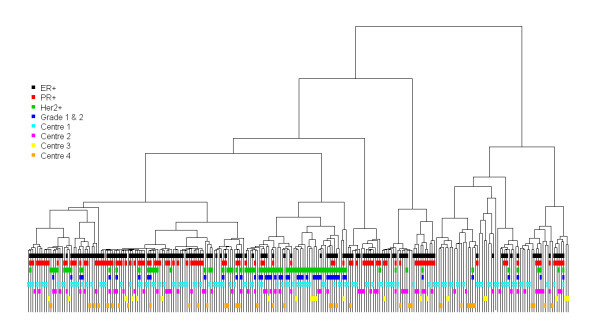
**RT-qPCR analysis: Clustering of 239 patients based on the 45 E^ΔΔCt ^target genes and considering the median Ct of the 6 reference genes as of the reference gene**.

Two hundred and thirty nine cases (82%) with adequate quality and quantity criteria and complete information for all genes were available for RT-qPCR statistical analysis. All samples had linear amplification of the TBP gene from 1/40 to 1/200 dilutions. NTC were greater than 40 in all cases. No amplification of albumin DNA was observed. High RT-qPCR efficiencies (> 90%) were found for each gene transcript. Mean Ct values were comprised between 20 and 27 for the majority of genes, and between 31 and 33 for KRT 5, KRT17, PTGS2, HTER, PROM1 and SERPINB5. Forty-four of the 45 selected genes were considered to be present in all samples, GSTM1 was undetectable (Ct > 35) in 22% of samples. Comparison of the mean Ct values of the 6 reference genes in the 239 cases showed that centre 4 exhibited significantly higher Ct values than the other 3 centres. No significant difference was observed between the 3 remaining centres after excluding centre 4 from the comparisons. Clustering analysis of the 45 target gene data obtained in 239 patients and expressed as E^ΔΔCt ^related to the median of the 6 references gene (similar to those expressed as E^ΔΔCt ^related to each of the 6 reference genes), highlighted ER+, PR+, HER2 +, and triple-negative clusters (Figure 3 andAdditional file 8). No centre effect was demonstrated.

## Discussion

This study stressed the importance of pre-analytical steps and demonstrated the feasibility and reliability of transcriptome and RT-QPCR analyses in the context of a randomised multicentre trial of neoadjuvant chemotherapy in breast cancer. Most published studies have concerned gene expression analyses in consecutive series of patients receiving neoadjuvant chemotherapy [12-15] and for review [16]. Only a few randomised trials with gene expression analyses have been conducted in the neoadjuvant setting [17-19]. In this study, ancillary laboratory studies were shared according to the experience of each laboratory associated with the clinical teams participating in the trial. These laboratories have obtained the "Hospital Molecular Genetics platforms for cancer" label from the French National Cancer Institute. Transcriptome analyses were centralised (Institut Curie translational research department), but RT-qPCR analyses were performed in two laboratories experienced in RT-qPCR quality control [6] and who set up common procedures for this study.

Based on our experience and on published data [20-24], we defined validation criteria for each type of analysis. Standard operating procedures were developed by the laboratories by testing selected modifications of the research protocols to the clinical setting and by including quality assurance measures. Limits of acceptability were defined at multiple steps including histological control of tumour cell percentage, and specific criteria for microarray and RT-qPCR quality controls. We used a minimal cutoff of 30% tumor cells to analyze the samples and we excluded 11% cases. There is no consensus on the best cut off for cellularity but our choice was in accordance with two major recent clinical trials; indeed, a cut off of 20% was used in the EORTC 10 994/BIG00-01 trial [16] or 30% in the Mindact trial [25]. While this is not generally performed, it would be of importance to examine the tumor characteristics of excluded cases, whatever the reason (low tumor cell content of unsufficient RNA quality or quantity) to better analyse potential biais generated in clinical trials with genomics performed on frozen samples. We observed in the R02 trial a higher proportion of lobular and low grade carcinoma in excluded cases. Around 80% of samples were retained for transcriptome and RT-qPCR analyses. RIN is generally considered to be a good tool to evaluate RNA quality and is used with a cut-off of 7 to select samples eligible for transcriptome analyses in multicentre clinical trials [25]. We used also additional quality control criteria (ratio of GAPDH and β-actin 3' to 5' probes, percent background signal, percent call and small and long cRNA fragment percentages).

Comparison of data from all centres showed that all transcriptome quality criteria, including RINs, were identical between centres, except that centre 4 presented the highest mean percentage of small cRNA and the lowest percentage of long cRNA. Interestingly, clustering in the overall population showed the relevant breast carcinoma phenotypes [26], but also demonstrated a "centre effect", probably due to the sampling or extraction methods used in centre 4. A statistical method described in Johnson et al. [27] was tested to handle this heterogeneity but this heterogeneity still remained. In addition, stability of this centre effect within clustering representation was also investigated and highlighted a great probability for centre 4 patients to be classified in the same cluster (see Additional file 2, 7 and 8).

In the present study, RIN appeared to be insufficient to adequately evaluate the quality of the samples. Samples with adequate RIN but poor quality cRNA affected the synthesis of full-length cDNA and hybridization efficiency with an impact on gene expression, as specific categories of genes may be most affected by RNA quality [22]**.**

The slightly different RNA extraction method used in centre 4 probably explained the differences in percentage of small and long cRNA. Handling of biopsies before freezing may also have been different. This procedure was optimized before initiation of the trial in each centre, but minor differences between centres that are difficult to control may have persisted. This problem is encountered in all multicentre prospective and retrospective, randomised and nonrandomised studies and should be taken into account when considering the heterogeneity of published transcriptome data. Strict acceptability criteria and quality controls were also applied to RT-qPCR analyses. The impact of RNA quality on RT-qPCR analyses was often not investigated in details in the literature, but the accuracy of gene expression analyses is clearly highly dependent on RNA quality [28-31]. A RIN value greater than 5 and a PCR product length up to 200 bp have been recommended to obtain reliable RT-qPCR results [29]. We used short PCR products and good quality RNA, as attested by RIN values above 6. The samples were also checked for the absence of PCR inhibitors. Moreover, sample-to-sample variation of PCR efficiency was corrected by using 8 points standard curves for each gene and each plate. Under these conditions, quantitative results were obtained for the 45 target genes. Higher Ct values were observed in centre 4, but with no impact on the expected classical breast cancer clustering. This suggests that the use of short amplicons and PCR normalization procedures allow obtaining reproducible RT-qPCR results with poorer quality RNA samples.

## Conclusions

Our data showed that even with strict quality criteria for RNA integrity assessment we observe a centre effect in the high throughput expression gene analysis. More stringent criteria are needed for high throughput analysis for clinical applications. However, well calibrated and standardized RT-qPCR allows multicentre analysis of genes transcripts with high accuracy in the clinical context.

## Conflict of interest statement

The authors declare that they have no competing interests.

## Authors' contributions

R02 working group: MM, FS, PdeC, FV, JLC, VS .BSZ, PB, NS, JMG, MCM and BA. MM, FS, PdeC, BA designed the clinical trial and ancillary biological trial. FS and PdeC coordinated the biological trial. JLC and VS realized RNA extractions. DG performed the microarray experiments. CTP, CB, SV performed RT-qPCR analysis. BSZ, PB, JMG, MCM performed and reviewed pathological diagnosis and pCR. FV performed all biostatistic and bioinformatic analysis. PdeC, FS, DG and FV drafted the manuscript.

All authors read and approved the manuscript

**Table 3:** Comparison of mean (standard deviation) Ct of reference genes analyzed by RT-qPCR, for the 4 centres

Reference Gene	Centre				All centres
		
	1	2	3	4	
**TFR**	23.85 (0.87)	24.09 (1.28)	24.23 (1.01)	26.30 (1.82)	24.34 (1.47)
**β-actin**	17.15 (0.58)	17.34 (1.19)	17.10 (0.62)	18.32 (0.88)	17.38 (0.94)
**TBP**	26.46 (0.53)	26.53 (1.18)	26.81 (0.47)	28.11 (1.10)	26.77 (1.03)
**GAPDH**	19.78 (0.94)	19.99 (1.27)	19.90 (0.77)	20.47 (1.05)	19.96 (1.07)
**RPLPO**	19.44 (0.60)	19.43 (1.06)	19.25 (0.78)	20.51 (0.93)	19.59 (0.91)
**GUS**	22.84 (0.71)	22.98 (1.23)	23.05 (0.76)	24.83 (1.33)	23.21 (1.21)

**p-value referring to ANOVA test for each reference gene, when excluding corresponding centre**					
**TFR**	< 0.001	< 0.001	< 0.001	0.144	< 0.001
**β-actin**	< 0.001	< 0.001	< 0.001	0.256	< 0.001
**TBP**	< 0.001	< 0.001	< 0.001	0.165	< 0.001
**GAPDH**	0.075	< 0.001	0.003	0.416	0.006
**RPLPO**	< 0.001	< 0.001	< 0.001	0.562	< 0.001
**GUS**	< 0.001	< 0.001	< 0.001	0.457	< 0.001

## Pre-publication history

The pre-publication history for this paper can be accessed here:

http://www.biomedcentral.com/1471-2407/11/215/prepub

## Supplementary Material

Additional file 1 **Supplemental Table 1. Details of genes analyzed by RT-qPCR**. This table listed the 45 genes that were analyzed by RTqPCR. Their symbols and biological pathways are also described. Click here for file

Additional file 2 **Supplemental Methods**. Complementary methods including microarrays data normalization, methods used for clustering and for the determination of clustering stability are described. Click here for file

Additional file 3 **Supplemental Table 2. Characteristics of available and excluded material for transcriptome analysis**. Clinical and pathological characteristics of excluded samples for transcriptome analysis are described. Comparison with the series of included samples is given (p-values). Click here for file

Additional file 4 **Supplemental Table 3. Characteristics of available and excluded material for RT-qPCR analysis**. Clinical and pathological characteristics of excluded samples for RT-qPCR analysis are described. Comparison with the series of included samples is given (p-values). Click here for file

Additional file 5 **Supplemental Table 4. Quantitative and qualitative characteristics of cRNA for transcriptome analysis median [min - max]**. Median yield of cRNA and median percentage of small and long cRNA are given for samples of each centre. Click here for file

Additional file 6 **Supplemental Table 5. Quantitative characteristics of GeneChip Array performance: median [min - max]**. Median background signal, median percentage present calls, median 3'/5' actin and GAPDH ratios are given for each centre. Click here for file

Additional file 7 **Supplemental Table 6: Stability results for clustering performed using transcriptomic data**. Stability of the clustering was assessed using a re-sampling approach as described in supplemental methods (Additional file 2). Click here for file

Additional file 8 **Supplemental Table 7. Stability results for clustering performed using RT-qPCR data**. Stability of the clustering was assessed using a re-sampling approach as described in supplemental methods (Additional file 2). Click here for file
