# Supplementary material for: Importance of pre-analytical steps for transcriptome and RT-qPCR analyses in the context of the phase II randomised multicentre trial REMAGUS02 of neoadjuvant chemotherapy in breast cancer patients
Source: BMC Cancer. 2011 Jun 1;11:215. doi: 10.1186/1471-2407-11-215 (PMC3126791; doi:10.1186/1471-2407-11-215)
Supplement: Additional file 1 — Supplemental Table 1. Details of genes analyzed by RT-qPCR. This table listed the 45 genes that were analyzed by RTqPCR. Their symbols and biological pathways are also described. [file 1471-2407-11-215-S1.PDF]

Additional files

Table S1: Details of genes analyzed by RT-qPCR

Gene symbol	Alternate symbol	Gene definition	Cellular Pathways
**ERBB2**	*c-erb B2*	*V-erb-b2 erythroblastic leukemia viral oncogene homolog 2*	**Growth factor receptors**
**GRB7**		*Growth factor receptor-bound protein 7*	
**ERBB4**	c-erb B4	V-erb-a erythroblastic leukemia viral oncogene homolog 4	
**ISR1**		*Insulin receptor substrate 1*	
**ESR1**	*ER alpha*	*Estrogen receptor 1 (alpha)*	**Estrogen receptors**
**PGR**	*PR*	*Progesterone receptor*	
**BCL2**		*B-cell leukemia 2 oncogene*	
**SCUBE2**		*Signal peptide, CUB domain, EGF-like 2*	
**ESR2**	*ER beta*	*Estrogen receptor 2 (beta)*	
**GATA3**		*GATA binding protein 3*	
**FOXA1**		*Forkhead box A1*	
**TFF1**	*PS2*	*Trefoil factor 1*	
**MKI67**	K*i-67*	*Proliferation-related Ki-67 antigen*	**Proliferation**
**AURKA**	*STK6, STK15*	*Aurora kinase A*	
**BIRC5**	*survivin*	*Baculoviral IAP repeat-containing 5*	
**CCNB1**		*Cyclin B1*	
**MYBL2**		*V-myb myeloblastosis viral oncogene homolog (avian)-like 2*	
**E2F1**	*RBBP3, RBP3*	*E2F transcription factor 1*	
**TOP2A**		Topoisomerase II, alpha	
**TTK**	PYT	TTK protein kinase	
**KRT5**		Keratin 5	**Basal component**
**KRT17**		Keratin 17	
**SERPINB5**		Maspin	
**TRIM29**		Tripartite motif-containing 29	
**KIT**	c-KIT	Kit oncogene	
**MMP11**	stromelysin 3	Matrix metallopeptidase 11	**Proteases**
**CTSL2**	CATL2	Cathepsin L2	
**MMP9**		Matrix metallopeptidase 9	
**GSTM1**		Glutathione S-transferase M1	**Treatment response**
**TUBB**		*Tubulin, beta*	
**MAPT**	*tau*	*Microtubule-associated protein tau*	
**FRAP1**	*mTOR*	*FK506 binding protein 12-rapamycin associated protein 1*	
**CD44**		*CD44 antigen*	**Stem cells**
**CD24**		*CD24 antigen*	
**PROM1**	*CD133*	*Prominin 1*	
**BBC3**	*PUMA*	*BCL-2 binding component 3*	**P53, apoptose**
**CDKN1A**	*p21*	*Cyclin-dependent kinase inhibitor 1A*	
**CDKN2A**	*p16*	*Cyclin-dependent kinase inhibitor 2A*	
**PTGS2**	*COX2*	*Prostaglandin-endoperoxide synthetase 2*	**Angiogenesis**
**VEGF**		*Vascular endothelial growth factor*	
**HIF1A**		*Hypoxia-inducible factor, alpha subunit*	
**CD68**		*CD68 antigen*	**Others**
**BAG1**		*BCL2-associated athanogene*	
**TERT**	*hTERT*	*Telomere reverse transcriptase*	
**BRCA1**		*Breast Cancer 1*	
**BRCA2**		*Breast Cancer*	
