# Supplementary material for: Importance of pre-analytical steps for transcriptome and RT-qPCR analyses in the context of the phase II randomised multicentre trial REMAGUS02 of neoadjuvant chemotherapy in breast cancer patients
Source: BMC Cancer. 2011 Jun 1;11:215. doi: 10.1186/1471-2407-11-215 (PMC3126791; doi:10.1186/1471-2407-11-215)
Supplement: Additional file 2 — Supplemental Methods. Complementary methods including microarrays data normalization, methods used for clustering and for the determination of clustering stability are described. [file 1471-2407-11-215-S2.PDF]

Additional files:

SUPPLEMENTARY METHODS

1 - Transcriptome and RT-qPCR analysis

Comparison of reference genes CT means
Comparisons of reference genes Ct means between each of the four centres were performed using ***aov*** function of the ***stats*** package.

Microarrays data normalization
Microarrays data were normalized together (all centres were normalized together) using the ***gcrma*** function of the ***gcrma*** package (release 2.18.0).

Inter-Quartile Range
Inter-Quartile Range of a probe set P was defined as the difference between the third quartile and the first quartile. Calculation was done using the ***quantile*** function of the ***stats*** package.

Clustering on RT-qPCR and transcriptome data
Clustering on RT-qPCR and transcriptomic data were performed using ***hclust*** function of the ***stats*** package. Distances matrix (similarities measures) were expressed as (1-CM)/2. For RT-qPCR, CM (Correlation Matrix) was defined as a 239 x 239 matrix corresponding to the Spearman correlations values between the 239 patients, based on the 45 target genes $E^{\Delta\Delta Ct}$ values. For transcriptomic data, CM was defined as a 226 x 226 matrix corresponding to the Spearman correlations values between the 226 patients, based on the 5000 probe sets having the highest inter-quartile ranges values.

2 - Cluster stability
As pointed-out by Giancarlo et al.(*Giancarlo, R., D. Scaturro, and F. Utro, Computational cluster validation for microarray data analysis: experimental assessment of Clest, Consensus Clustering, Figure of Merit, Gap Statistics and Model Explorer. BMC Bioinformatics, 2008.* ***9****: p. 462.*), inferring cluster stability in microarray datasets is a fundamental task However, for large data sets (for example for a clustering made using 5000 probe sets), computation of stability measures may be non-trivial and time consuming (*Kraus, J.M. and H.A. Kestler, A highly efficient multi-core algorithm for clustering extremely large datasets. BMC Bioinformatics.* ***11****: p. 169.*). In addition, in our study, identification of a centre 4 effect encouraged us to investigate more precisely the ability of centre 4 patients to be classified in the same cluster. To this aim, we focused on the stability of this specific centre effect in both clusterings made using transcriptomic or RT-qPCR data.
To this aim, a re-sampling approach was proposed. From the original data set, we defined 1000 hierarchical clusterings by randomly removing 1000 times, 5% of the total number of variables (i.e. 250 of the 5000 probe sets for transcriptomic data, and 2 of the 45 target genes for RT-qPCR data). Then, among these 1000 clusterings thus defined, we evaluated for a number of clusters ranging from 2 to 6, the mean proportion of patients of each centre included in the different clusters. In order to investigate the ability of centres to define a specific cluster, the ratio between the number of patients of the same centre in the same cluster, and the cluster size was also computed. As previously defined, we used Spearman correlations as similarity measures and a Ward algorithm as the agglomerative criterion. Results were given in table 4s and 5s for clusterings with transcriptome and RT-qPCR, respectively.
