# Supplementary material for: Importance of pre-analytical steps for transcriptome and RT-qPCR analyses in the context of the phase II randomised multicentre trial REMAGUS02 of neoadjuvant chemotherapy in breast cancer patients
Source: BMC Cancer. 2011 Jun 1;11:215. doi: 10.1186/1471-2407-11-215 (PMC3126791; doi:10.1186/1471-2407-11-215)
Supplement: Additional file 3 — Supplemental Table 2. Characteristics of available and excluded material for transcriptome analysis. Clinical and pathological characteristics of excluded samples for transcriptome analysis are described. Comparison with the series of included samples is given (p-values). [file 1471-2407-11-215-S3.PDF]

Additional files

Table S2: Characteristics of available and excluded material for transcriptome analysis

	Available material (N=226)	Excluded material (N=327-226=101)	p-value*
Variables			
Mean of tumor size (mm)	51.50	52.66	0.659
% of ER+	61%	63%	0.789
% of PR+	42%	37%	0.431
% of HER2+	36%	37%	0.791
% of Grade <3	43%	58%	0.018
Histological type			
ductal carcinoma	88%	79%	0.051
lobular carcinoma	7%	18%	0.003
others	5%	3%	0.923

* p-values associated to tests for comparing two observed means (for tumor size) and proportions (for ER+, PR+, HER+, Grade, histologycal type)
