# Supplementary material for: Importance of pre-analytical steps for transcriptome and RT-qPCR analyses in the context of the phase II randomised multicentre trial REMAGUS02 of neoadjuvant chemotherapy in breast cancer patients
Source: BMC Cancer. 2011 Jun 1;11:215. doi: 10.1186/1471-2407-11-215 (PMC3126791; doi:10.1186/1471-2407-11-215)
Supplement: Additional file 4 — Supplemental Table 3. Characteristics of available and excluded material for RT-qPCR analysis. Clinical and pathological characteristics of excluded samples for RT-qPCR analysis are described. Comparison with the series of included samples is given (p-values). [file 1471-2407-11-215-S4.PDF]

Additional files

Table S3: Characteristics of available and excluded material for RT-qPCR analysis

	Available material (N=239)	Excluded material (N=327-239=88)	p-value
Mean of tumor size (mm)	51.75	52.14	0.884
% of ER+	61%	64%	0.591
% of PR+	41%	38%	0.621
% of HER2+	35%	41%	0.323
% of Grade <3	44%	57%	0.044
Histological type			
ductal carcinoma	87%	80%	0.103
lobular carcinoma	7%	18%	0.004
others	6%	2%	0.158

* p-values associated to tests for comparing two observed means (for tumor size) and proportions (for ER+, PR+, HER+, Grade, histologycal type)
