# Supplementary material for: Importance of pre-analytical steps for transcriptome and RT-qPCR analyses in the context of the phase II randomised multicentre trial REMAGUS02 of neoadjuvant chemotherapy in breast cancer patients
Source: BMC Cancer. 2011 Jun 1;11:215. doi: 10.1186/1471-2407-11-215 (PMC3126791; doi:10.1186/1471-2407-11-215)
Supplement: Additional file 5 — Supplemental Table 4. Quantitative and qualitative characteristics of cRNA for transcriptome analysis median [min - max]. Median yield of cRNA and median percentage of small and long cRNA are given for samples of each centre. [file 1471-2407-11-215-S5.PDF]

Additional files

Table S4: Quantitative and qualitative characteristics of cRNA for transcriptome analysis **median** [min - max]

**Characteristics**	**Centre**				**All centres**
	**1**	**2**	**3**	**4**	
**Number of samples**	108	62	19	37	226
**Median yield of cRNA in µg***	**74** [43 - 95]	**67** [32 - 102]	**60** [42 - 80]	**36** [13 - 77]	**68** [13 - 102]
**Median percentage of small cRNA (<500nt) ****	**24** [9 - 35]	**27** [19 - 39]	**27** [22 – 35]	**32** [26 - 45]	**26** [9 - 45]
**Median percentage of long cRNA (>1500nt) ****	**44** [27 - 70]	**40** [25 - 52]	**39** [27 - 45]	**28** [18 - 39]	**41** [18 - 70]

* 20µg are required for the target preparation

** U133plus2.0 hybridization = less than 39% of small fragments and more than 20% of long fragments
