# Supplementary material for: Importance of pre-analytical steps for transcriptome and RT-qPCR analyses in the context of the phase II randomised multicentre trial REMAGUS02 of neoadjuvant chemotherapy in breast cancer patients
Source: BMC Cancer. 2011 Jun 1;11:215. doi: 10.1186/1471-2407-11-215 (PMC3126791; doi:10.1186/1471-2407-11-215)
Supplement: Additional file 6 — Supplemental Table 5. Quantitative characteristics of GeneChip Array performance: median [min - max]. Median background signal, median percentage present calls, median 3'/5' actin and GAPDH ratios are given for each centre. [file 1471-2407-11-215-S6.PDF]

Addittional files

Table S5: Quantitative characteristics of GeneChip Array performance: **median** [min - max]

**Characteristics**	**Centre**				**All centres**
	**1**	**2**	**3**	**4**	
**Number of samples**	108	62	19	37	226
**Median background signal (µg)**	**62.20** [42.87 – 184.99]	**57.08** [43.87 – 84.64]	**61.56** [45.91 – 73.15]	**59.51** [44.33 – 85.12]	**59.66** [42.87 – 184.99]
**Median % Present calls**	**50.90** [35.26 – 57.05]	**51.77** [44.44 – 56.54]	**49.02** [46.06 – 51.63]	**44.35** [34.27 – 49.96]	**50.04** [34.27 – 57.05]
**Median 3'/5' Actin ratio**	**2.31** [1.47 – 19.58]	**2.45** [1.48 – 69.62]	**1.81** [1.42 – 3.15]	**1.82** [1.41 – 2.95]	**2.24** [1.41 – 69.62]
**Median 3'/5' GAPDH ratio**	**1.27** [0.99 – 2.90]	**1.38** [1.00 – 83.65]	**1.21** [1.01 – 1.56]	**1.10** [0.93 – 1.47]	**1.27** [0.93 – 83.65]
