# Supplementary material for: Importance of pre-analytical steps for transcriptome and RT-qPCR analyses in the context of the phase II randomised multicentre trial REMAGUS02 of neoadjuvant chemotherapy in breast cancer patients
Source: BMC Cancer. 2011 Jun 1;11:215. doi: 10.1186/1471-2407-11-215 (PMC3126791; doi:10.1186/1471-2407-11-215)
Supplement: Additional file 7 — Supplemental Table 6: Stability results for clustering performed using transcriptomic data. Stability of the clustering was assessed using a re-sampling approach as described in supplemental methods (Additional file 2). [file 1471-2407-11-215-S7.PDF]

Additional files

Table S6 Stability results for clustering performed using transcriptomic data.

a. Mean proportions of patients in the different clusters according to the four different centres.

	Hierarchical Clustering with 2 clusters		3 clusters			4 clusters				5 clusters					6 clusters					
	**1**	**2**	**1**	**2**	**3**	**1**	**2**	**3**	**4**	**1**	**2**	**3**	**4**	**5**	**1**	**2**	**3**	**4**	**5**	**6**
**Centre 1**	0.80	0.20	0.77	0.00	0.23	0.36	0.00	0.41	0.23	0.35	0.00	0.41	0.23	0.01	0.22	0.00	0.30	0.26	0.22	0.00
**Centre 2**	0.87	0.13	0.86	0.04	0.10	0.41	0.04	0.44	0.11	0.41	0.02	0.43	0.12	0.02	0.26	0.03	0.29	0.30	0.11	0.01
**Centre 3**	0.21	0.79	0.20	0.71	0.09	0.13	0.71	0.07	0.09	0.13	0.29	0.07	0.05	0.46	0.10	0.27	0.06	0.04	0.06	0.47
**Centre 4**	0.01	**0.99**	0.00	**0.98**	0.02	0.00	**0.98**	0.00	0.02	0.00	**0.74**	0.00	0.00	0.26	0.00	**0.72**	0.00	0.00	0.01	0.27

The proportions of centre 4 patients classified in the same cluster ranged from 0.99 to 0.72, for a number of clusters ranging from 2 to 6. These proportions are quite higher than those of the others centres, suggesting that whatever the number of optimal clusters, patients of centre 4 are more likely to be classified in the same cluster.

b. Mean ratio between numbers of patients in the different clusters and the size of each cluster.

	Hierarchical Clustering with 2 clusters		3 clusters			4 clusters				5 clusters					6 clusters					
	**1**	**2**	**1**	**2**	**3**	**1**	**2**	**3**	**4**	**1**	**2**	**3**	**4**	**5**	**1**	**2**	**3**	**4**	**5**	**6**
**Centre 1**	0.60	0.25	0.59	0.00	0.75	0.59	0.00	0.62	0.74	0.58	0.00	0.62	0.76	0.05	0.55	0.00	0.64	0.57	0.74	0.03
**Centre 2**	0.37	0.10	0.38	0.04	0.20	0.37	0.04	0.35	0.20	0.37	0.05	0.35	0.21	0.05	0.41	0.05	0.33	0.41	0.20	0.04
**Centre 3**	0.03	0.19	0.03	0.26	0.04	0.04	0.26	0.03	0.04	0.05	0.15	0.03	0.03	0.43	0.04	0.15	0.03	0.02	0.04	0.43
**Centre 4**	0.00	**0.46**	0.00	**0.70**	0.01	0.00	**0.70**	0.00	0.02	0.00	**0.80**	0.00	0.00	0.48	0.00	**0.81**	0.00	0.00	0.02	0.50

In the second cluster, the number of centre 4 patients as compared to the total number of patients in the same cluster ranged from 0.46 to 0.81, for a number of clusters ranging from 2 to 6. These results suggested that the contribution of centre 4 patients for the definition of cluster 2 ranged between 46% and 81%.
