# Supplementary material for: Importance of pre-analytical steps for transcriptome and RT-qPCR analyses in the context of the phase II randomised multicentre trial REMAGUS02 of neoadjuvant chemotherapy in breast cancer patients
Source: BMC Cancer. 2011 Jun 1;11:215. doi: 10.1186/1471-2407-11-215 (PMC3126791; doi:10.1186/1471-2407-11-215)
Supplement: Additional file 8 — Supplemental Table 7. Stability results for clustering performed using RT-qPCR data. Stability of the clustering was assessed using a re-sampling approach as described in supplemental methods (Additional file 2). [file 1471-2407-11-215-S8.PDF]

Additional files

Table S7: Stability results for clustering performed using RT-qPCR data.

a. Mean proportions of patients in the different clusters according to the four different centres.

	**Hierarchical Clustering with**																			
	**2 clusters**		**3 clusters**			**4 clusters**				**5 clusters**					**6 clusters**					
	**1**	**2**	**1**	**2**	**3**	**1**	**2**	**3**	**4**	**1**	**2**	**3**	**4**	**5**	**1**	**2**	**3**	**4**	**5**	**6**
**Centre 1**	0.42	0.58	0.36	0.36	0.28	0.26	0.29	0.25	0.20	0.25	0.23	0.22	0.20	0.10	0.25	0.20	0.19	0.19	0.14	0.03
**Centre 2**	0.33	0.67	0.26	0.47	0.27	0.15	0.41	0.23	0.21	0.14	0.34	0.21	0.20	0.11	0.13	0.29	0.19	0.19	0.16	0.04
**Centre 3**	0.44	0.56	0.37	0.46	0.17	0.20	0.43	0.13	0.24	0.18	0.37	0.14	0.17	0.14	0.17	0.33	0.14	0.12	0.19	0.05
**Centre 4**	0.35	0.65	0.31	0.58	0.11	0.26	0.53	0.11	0.10	0.24	0.29	0.15	0.11	0.21	0.24	0.16	0.12	0.09	0.08	0.31

There were no outstanding differences between the proportions of patients from centre 4, within the different clusters

b. Mean ratio between numbers of patients in the different clusters and the size of each cluster.

	**Hierarchical Clustering with**																			
	**2 clusters**		**3 clusters**			**4 clusters**				**5 clusters**					**6 clusters**					
	**1**	**2**	**1**	**2**	**3**	**1**	**2**	**3**	**4**	**1**	**2**	**3**	**4**	**5**	**1**	**2**	**3**	**4**	**5**	**6**
**Centre 1**	0.53	0.46	0.54	0.40	0.57	0.57	0.38	0.57	0.49	0.57	0.40	0.54	0.52	0.31	0.58	0.42	0.52	0.54	0.48	0.15
**Centre 2**	0.23	0.30	0.21	0.30	0.30	0.18	0.30	0.30	0.30	0.18	0.33	0.28	0.31	0.19	0.18	0.35	0.30	0.31	0.30	0.08
**Centre 3**	0.10	0.08	0.10	0.10	0.06	0.08	0.10	0.05	0.11	0.07	0.12	0.06	0.08	0.08	0.07	0.13	0.07	0.06	0.12	0.04
**Centre 4**	0.14	0.16	0.15	0.20	0.07	0.17	0.22	0.08	0.10	0.18	0.15	0.12	0.09	0.42	0.17	0.10	0.11	0.09	0.10	0.73

The number of centre 4 patients as compared to the total number of patients was quite homogeneous in each cluster.
